# The effect of aspirin on circulating netrin-1 levels in humans is dependent on the inflammatory status of the vascular endothelium

**DOI:** 10.18632/oncotarget.21240

**Published:** 2017-09-23

**Authors:** Kerry Layne, Timothy Goodman, Albert Ferro, Gabriella Passacquale

**Affiliations:** ^1^ Department of Clinical Pharmacology, BHF Centre of Research Excellence, Cardiovascular Division, King’s College London, London, UK

**Keywords:** netrin-1, endothelium, immunisation, inflammation, cyclo-oxygenase

## Abstract

In atherosclerotic animal models, the cyclo-oxygenase (COX)-inhibitor aspirin counteracts downregulation of endothelial-derived netrin-1, thus reducing arterial inflammation. We here explored the effect of aspirin on netrin-1 in healthy subjects undergoing influenza immunisation, which is an established experimental model of inflammation-related endothelial dysfunction.

Our data showed that netrin-1 undergoes reduction (-29.25% from baseline; p=0.0017) in the presence of endothelial activation (VCAM-1 rose by 9.98% 2-days post-vaccination; p=0.0022). Aspirin counteracted vaccine-induced endothelial activation and reduction of netrin-1 in a dose-dependent manner (-3.06% and -17.03% from baseline at a dose of 300mg and 75mg respectively; p=0.0465 and p>0.05 vs untreated). Clopidogrel, which was used as a comparator due to its similar anti-platelet activity, also reduced endothelial activation but, unlike aspirin, enhanced netrin-1 levels (+20.96% from baseline; p=0.0033 vs untreated). A correlation analysis incorporating cytokines, hs-CRP, VCAM-1, TXB_2_ and PGE_2_, showed that changes in netrin-1 were directly related to PGE_2_ variations only (r=0.6103; p=0.0002). In a separate population of 40 healthy unimmunised volunteers, 28-day treatment with aspirin 300mg reduced netrin-1 (-18.76% from baseline; p=0.0012) without affecting endothelial markers or hs-CRP; as expected, aspirin suppressed TXB_2_ and PGE_2_. Netrin-1 and PGE_2_ levels were directly related (r=0.358; p=0.0015), but other parameters including TXB_2_, hs-CRP and endothelial markers, were not.

In conclusion, aspirin counteracts downregulation of netrin-1 following endothelial dysfunction due to its anti-inflammatory effect on the activated endothelium. However, inhibition of COX-dependent prostanoids negatively modulates netrin-1 synthesis in healthy subjects, and this could give rise to aspirin-dependent reduction in netrin-1 under steady state conditions.

## INTRODUCTION

Netrins are a class of laminin-like proteins, which were initially isolated within the central nervous system and identified as regulators of embryonic axonal guidance. Netrin-1, by far the best-characterised member of the group, has subsequently been found to have a wide spectrum of regulatory roles in numerous pathological conditions, given its broad expression in inflammatory, vascular and tumour cell types and ability to control their survival, apoptosis and migration. Netrin-1 has become particularly relevant within the field of oncology, where it has been shown to be of both diagnostic and prognostic value in many cancer subtypes [1, 2], and there is much interest in the development of therapeutic antibodies that interfere with netrin-1-dependent pathways as a potentially novel chemotherapeutic approach [3]. Netrin-1 is also emerging as a therapeutic target in cardiovascular disease [4], having been shown in pre-clinical studies to modulate atherogenesis via the control of arterial inflammation [5], as well as exerting cardio- and renoprotective actions [6, 7]; and in this context, enhancing netrin-1 signalling may be desirable.

To date, there has been limited characterisation of the *in vivo* pathways that regulate netrin-1 expression in humans, which may compromise the development of targeted therapeutic strategies to achieve either a stimulatory or an inhibitory effect on the synthesis of this important molecule in various clinical settings. Moreover, different isoforms of netrin-1 are produced which are differently regulated. Our previous work on the vascular endothelium [8] has showed that NF-κB activation, as induced by pro-inflammatory stimuli, up-regulates the synthesis of the nuclear isoform of netrin-1, which is involved in cell survival [9, 10]. On the contrary, a reduction in the secretion of the endothelial-derived full-length isoform of netrin-1, which protects against arterial inflammation, occurs in response to pro-inflammatory/proatherogenic stimuli as a consequence of endothelial damage. Administration of aspirin counteracted the downregulation of netrin-1 within the vascular endothelium in a murine model of atherosclerosis, leading to a beneficial action in terms of the inflammatory plaque component [8].

In the current study, we investigated the effect of endothelial dysfunction on the circulating levels of netrin-1 in humans. Endothelial dysfunction is a systemic perturbation of vascular homeostasis characterised by impaired endothelium-dependent vasodilation and an associated state of endothelial activation, which is not only triggered by pathological stimuli in the context of cardiovascular diseases but also occurs as part of physiological host defence mechanisms during inflammation [11, 12]. In keeping with this, vaccine administration is regarded as a valid human experimental model to study cardiovascular physiology, since the mild systemic inflammation triggered by immunisation induces a transient endothelial dysfunction [13, 14]. In this context, the therapeutic potential of the cyclo-oxygenase (COX) inhibitor, aspirin, in modulating netrin-1 synthesis was studied.

## RESULTS

### Endothelial dysfunction results in reduced circulating levels of netrin-1

**Figure 1:**
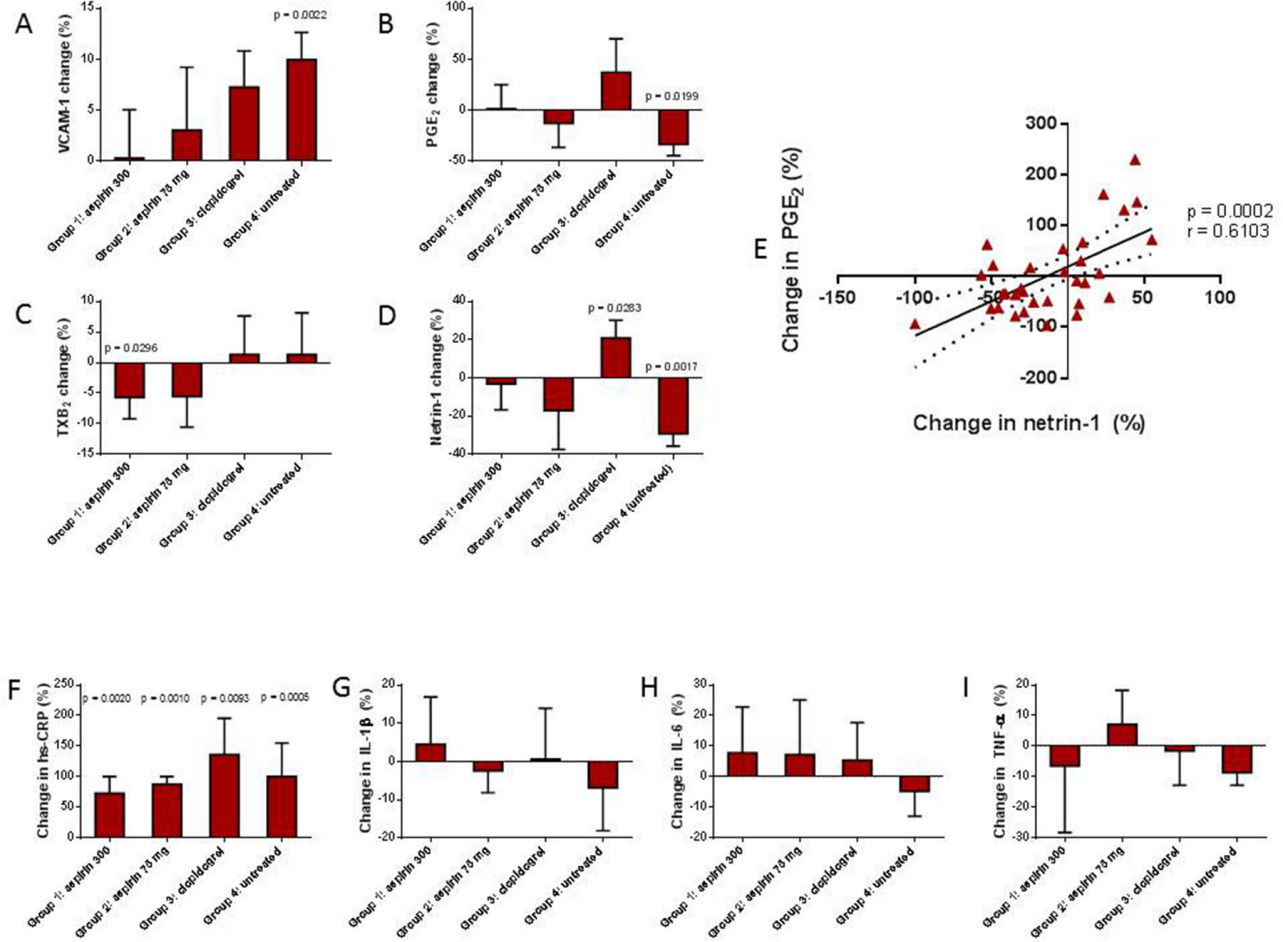
Influenza immunisation induced a rise in the endothelial marker VCAM-1 **(Panel A)**, with a concomitant reduction in serum prostaglandin E2 (PGE2; **Panel B**) and netrin-1 **(Panel D)**. All anti-platelet drug regimes abolished these effects. Serum thromboxane B_2_ (TXB_2_) was reduced in those taking aspirin 300 mg **(Panel C)**. There was a positive correlation between change in netrin-1 and PGE_2_ **(Panel E)**. High-sensitivity C-reactive protein (hs-CRP; **Panel F**) was elevated in all participants, whilst there was no significant reduction in pro-inflammatory cytokines, interleukin 1 beta (IL-1β; **Panel G**), interleukin-6 (IL-6; **Panel H**), and tumour necrosis factor alpha (TNF-α; **Panel I**). Values are reported as either mean with SEM or median and IQR. P values are shown where data are significant.

Immunisation has previously been reported to induce vascular dysfunction in healthy volunteers [11, 15]. In accordance with these data, the present study demonstrated an increase in serum level of VCAM-1, which rose from a baseline level of 493.7 ± 44.34 ng/ml to 542.3 ± 49.60 ng/ml post-influenza immunisation, in participants untreated with anti-platelet agents (Group 4; p=0.0022 vs baseline). In the same subjects, circulating levels of netrin-1 underwent a reduction of approximately 30%, from 311.9 (IQR 248.0 – 316.3) pg/ml at baseline to 220.2 (IQR 202.2 – 287.7) pg/ml post-immunisation (p=0.0017; Figure 1).

All anti-platelet drug regimes tested suppressed this rise in VCAM-1, although the 300 mg dose of aspirin appeared to be more effective at doing so than both the 75 mg dose of aspirin and clopidogrel (Figure 1). Aspirin counteracted the suppression of netrin-1 post-immunisation in a dose-dependent manner (% change from baseline in Groups 1 and 2 were -3.06 and -17.03 respectively; p=0.0465 and p>0.05 vs untreated), whilst a significant increase in the level of netrin-1 was observed in the clopidogrel group (20.96% change from baseline; p=0.0033 vs untreated) (Figure 1).

Levels of serum TXB_2_, a marker of platelet activation, which is widely used as an indicator of aspirin responsiveness, fell significantly, from 3416.0 ± 285.7 ng/ml at baseline to 3130.0 ± 244.0 ng/ml post-immunisation, in the group receiving aspirin 300 mg for 28 days (p=0.0296). There was a reduction in TXB_2_ also in those participants on aspirin 75 mg for 48 hours, however this did not reach statistical significance (p=0.2103). Post-immunisation TXB_2_ levels remained stable in the untreated participants, reflecting a lack of pro-thrombotic effect of influenza immunisation; also, clopidogrel did not influence serum TXB_2_ levels, in agreement with prior findings showing no effect of clopidogrel on TXB_2_ when administered to healthy volunteers in the absence of pro-thrombotic stimuli [16] (Figure 1). On the contrary, levels of PGE_2_, which is known to have a protective effect on the vascular endothelium under steady state conditions [17–20] and whose concentration is regulated by a balance between its COX-dependent synthesis and degradation rates [21], fell from 320.9± 55.04 pg/ml to 189.5 ±30.37 pg/ml in the untreated participants (p=0.0199 vs baseline). PGE_2_ reduction post-immunisation was attenuated by aspirin treatment, whilst in the clopidogrel-treated group, a non-significant trend towards an increase in PGE_2_ was observed (Figure 1). Overall, post-immunisation changes in PGE_2_ and netrin-1 followed the same pattern in the different groups (Figure 1).

### Netrin-1 levels are directly associated with PGE_2_ production

In a multiple regression analysis of the correlation between netrin-1 levels and all other study parameters, including markers of endothelial function, TXB_2_ and PGE_2_, as well as previously measured and reported parameters (hs-CRP, cytokines, P-selectin levels and monocyte cell subset count), we found that changes in netrin-1 levels in response to immunisation either with or without anti-platelet treatment were linearly and directly related with changes in PGE_2_ concentration only (r=0.6103; p=0.0002; Figure 1).

**Figure 2:**
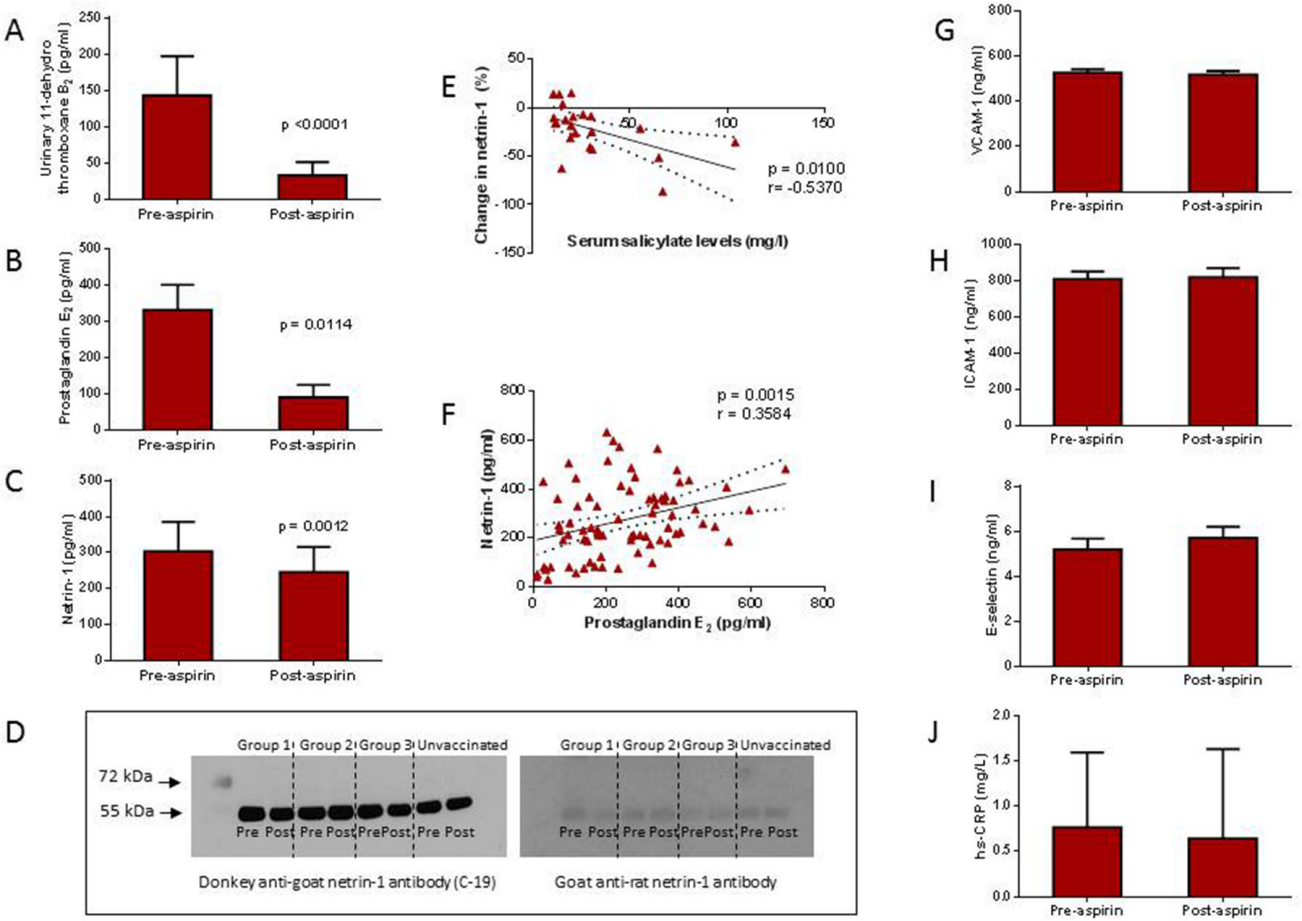
In healthy volunteers, aspirin 300 mg administered for 28 days led to a reduction in serum prostaglandin E2 (PGE2; **Panel B**) and netrin-1 **(Panel C)**, as well as thromboxane B2 (TXB2; **Panel A**). The reduction in netrin-1 was associated with increased serum salicylate levels **(Panel E)**, while there was a positive correlation between netrin-1 and PGE_2_ levels **(Panel F)**. There was no change in markers of endothelial dysfunction, vascular cell adhesion molecule-1 (VCAM-1; **Panel G**), intracellular adhesion molecule (ICAM-1; **Panel H**), or E-selectin (**Panel I**). High-sensitivity C-reactive protein (hs-CRP) levels remained unchanged post treatment **(Panel J)**. Western blotting was performed to identify both the full length, 70 kDa netrin-1 protein and the truncated, 55 kDa netrin-1 protein, but only the truncated isoform was detected **(Panel D)**.

Previous experimental data have demonstrated that netrin-1 inhibits PGE_2_ production via suppression of COX-2 expression. However, in contrast, our data showed a direct and positive relationship between these two parameters. In order to further delineate their mutual interaction, we measured netrin-1 levels in the cohort of healthy subjects taking 28 days of treatment with 300 mg aspirin, which primarily targets the synthesis of COX-dependent eicosanoids. Drug treatment in this cohort, as expected, led to a significant reduction in both TXB_2_ and PGE_2_ levels (Figure 2). This latter fell from 331.9 ± 69.04 pg/ml at baseline to 89.99 ± 34.29 pg/ml post-aspirin therapy (p=0.0114). Serum VCAM-1 remained unchanged from baseline levels of 527.32 ± 15.46 ng/ml to 519.10±15.76 ng/ml post-treatment. ICAM-1 and E-selectin were also unchanged, indicating no effect of aspirin on the inflammatory status of the endothelium in these study participants. Levels of hs-CRP were also not affected (Figure 2).

Interestingly, there was a mild but consistent reduction in serum netrin-1, from a baseline level of 303.9 (IQR: 218.4 – 386.0) pg/ml to 246.9 (IQR: 193.2 – 316.2) pg/ml post-aspirin therapy (p=0.0012) (Figure 2). When combining baseline and post-treatment values of all study variables, a strong positive correlation was found between netrin-1 and PGE_2_ levels (r=0.3584; p=0.0015) only. The percentage change in netrin-1 was found to be negatively correlated with serum salicylate levels (r=-0.5370; p=0.0100), indicating that the reduction in netrin-1 was a consequence of aspirin therapy (Figure 2).

To characterise the netrin-1 isoform that was being measured in serum samples by ELISA, we performed Western blotting using a rat anti-netrin-1 antibody to specifically target the full length (70 kDa) protein and a goat anti-netrin-1 antibody against the truncated (55 kDa) protein. We were only able to detect the truncated isoform of netrin-1 in serum samples (Figure 2).

## DISCUSSION

Our prior work both *in vitro* and in a mouse model of atherosclerosis (8) have demonstrated that a dysfunctional endothelium markedly loses its ability to secrete netrin-1, and this compromises the integrity of the vascular barrier against inflammatory cell infiltration. The current clinical study confirms these experimental data, whilst offering insight into the biomolecular mechanisms that regulate the synthesis of vascular netrin-1 in humans under physiological conditions.

Endothelial activation in response to influenza immunisation was confirmed by the increased level of VCAM-1 observed in our subjects 48 hours post-immunisation, supporting the validity of influenza immunisation as an experimental model to study the relationship between endothelial dysfunction and circulating levels of netrin-1 in humans. In accordance with prior studies performed in several animal models of inflammation [8, 22, 23], we found that endothelial activation, as driven by mild inflammation, is paralleled by a reduction in the serum concentration of netrin-1 in healthy subjects. This effect was abolished by anti-platelet treatment independently of their mode of action or of their ability to suppress systemic inflammation. Indeed, we have previously reported the lack of efficacy of both aspirin and clopidogrel in modulating the rise in hs-CRP with this experimental model of inflammation [24]. Moreover, whilst aspirin 300 mg exerted the strongest inhibition on platelet activity, as evidenced by greater suppression of P-selectin (10) and TXB_2_ in response to immunisation than with clopidogrel, this latter was better at restoring netrin-1 levels post-immunisation. All anti-platelet agents exerted comparable effects in terms of suppression of the rise in VCAM-1 induced by immunisation. However, aspirin, which specifically inhibits COX activity, suppressed PGE_2_ levels and the degree of suppression of PGE_2_ was directly related to changes in netrin-1 levels in both studies.

Our data demonstrate that netrin-1 reduction, as occurs in the presence of a dysfunctional endothelium caused by immunisation, occurs in parallel with PGE_2_ reduction. On the other hand, pharmacological inhibition of PGE_2_ as induced by the COX-inhibitor aspirin, also produced a small but significant decrease in netrin-1 levels, in the absence of endothelial dysfunction. The TXA_2_-pathway does not appear to be involved in netrin-1 regulation, inasmuch as modulation of netrin-1, both in subjects receiving influenza immunisation and in those receiving high-dose aspirin for 28 days, was not related to changes in serum TXB_2_. Although we measured PGE_2_ here, we cannot exclude the possibility that additional COX-dependent endothelium-derived prostanoids, such as prostacyclin, that generally follow the same pattern of expression in healthy endothelium [25], may show a similar association with netrin-1 under these experimental conditions. However, considering the prior demonstrated modulatory effect of netrin-1 on COX-2 expression and PGE_2_ synthesis, it is intriguing to note the existence of a regulatory link between COX-activity, COX-dependent prostaglandin production, and netrin-1 that may have important functional and therapeutic implications.

Numerous studies have demonstrated that PGE_2_ is protective against endotoxin injury, and promotes endothelial barrier enhancement [17–20, 26, 27] as well as cell survival [28, 29]. The observed reduction in PGE_2_ in response to immunisation was abolished by all anti-platelet therapy regimes, likely due to their beneficial effect on the vascular endothelium that, in turn, preserved its ability to synthesise protective vasoactive molecules and/or reduced PGE_2_ consumption. Of note, there was a tendency towards an increase in PGE_2_ levels in those taking clopidogrel, which we attribute to the suppression of endothelial activation driven by clopidogrel, without the additional effect of COX-inhibition leading to reduced PGE_2_ production which occurs with aspirin therapy. The anti-inflammatory action exerted by anti-platelet agents on the vasculature that was seen in this study could partly be ascribed to their ability to suppress expansion of the pro-inflammatory monocytes that we have previously demonstrated in this human model of inflammation [24]. However, a few differences were noted in this clinical study compared to our previous pre-clinical work using the same anti-platelet agents [8]. The superior effect of aspirin over clopidogrel in counteracting netrin-1 reduction in atherosclerotic mice was not observed in this clinical study; rather, clopidogrel demonstrated a better ability to maintain circulating levels of netrin-1 in the presence of endothelial dysfunction in humans. This discrepancy may be attributable to the distinct modulation of the tested anti-platelet agents on PGE_2_ production, which has emerged as an important factor implicated in the regulation of netrin-1 in human physiological settings. Moreover, we were only able to detect the truncated isoform of netrin-1 in the blood of our subjects, whilst our previous *in vitro* experiments on endothelial cells reported a specific action of aspirin exerted on the full-length secreted isoform of the protein through an epigenetic modification of chromatin. Whether lack of expression of full-length netrin-1 is due to the phenomenon of protein truncation occurring in human serum cannot be excluded. Of note, in revising our prior published *in vitro* tests performed on human endothelial cells, we noted that stimulation with aspirin alone (in the absence of TNF as a co-stimulatory agent) showed a tendency, albeit not statistically significant, towards a reduction in netrin-1 release in serum supernatants by vascular cells compared to untreated ones. In light of the new data that has emerged from the current clinical study, it is likely that the aspirin-induced PGE_2_ reduction, which we reported in the aforementioned *in vitro* set of experiments, have accounted for a subtle difference in netrin-1 that only became evident and statistically relevant in the *in vivo* human studies here described.

In conclusion, our data suggest that circulating netrin-1 levels are directly modulated by changes in the status of activation of the vascular endothelium as well by changes in COX-dependent vasoactive prostanoids, such as PGE_2_, in healthy subjects. Given the emerging role of netrin-1 in cardiovascular disease and as an oncological target, further delineation of the pathways that control netrin-1 production under both physiological and pathological conditions, may allow development of useful and novel therapies based on netrin-1 modulation.

## MATERIALS AND METHODS

### Participant recruitment

This was a post-hoc investigation conducted on a total of 76 healthy volunteer subjects, previously recruited at Guy’s and St Thomas’ Hospitals, London, who underwent two separate clinical studies and for whom serum samples were still available. All participants were aged 18 years or older (median: 31 years; IQR 25 - 38), had no significant medical history, were not taking regular medications, and had not taken anti-platelet or anti-inflammatory drugs in addition to the study medications described below.

36 of these subjects were participants in a study previously reported by our group [24], in which influenza immunisation was used as an experimental model of mild inflammation to evaluate the effect of different anti-platelet therapy regimes on a number of inflammatory biomarkers, including cytokines, monocyte phenotype, and high-sensitivity C-reactive protein (hs-CRP). Serum samples were collected before and 48 hours after receiving the seasonal influenza vaccine [24], and in the presence of one of the following treatments: aspirin 300 mg once daily (Group 1; n = 9); aspirin 75 mg once daily (group 2; n = 9); or clopidogrel with an initial loading dose of 300 mg followed by a further dose of 75 mg 24 h later (Group 3; n = 9) - see Table 1. Immunised participants on no anti-platelet treatment (Group 4, n=9) served as controls. For the purpose of the current study, stored serum samples were used to measure netrin-1, vascular cell adhesion molecule-1 (VCAM-1, as a marker of endothelial activation), prostaglandin E_2_ (PGE_2_, which has been demonstrated to exert vascular protective effects [18, 27] and is known to be regulated by netrin-1 via suppression of COX-2 [30], and thromboxane B_2_ (TXB_2_), as indicators of response to COX-inhibition [16]. Data were pooled with previously collected information, including hs-CRP, P-selectin and cytokine levels and monocyte phenotype as previously published [16].

**Table 1:** Baseline characteristics of study population

	Gender (n)	Age (years)	SBP (mmHg)	DBP (mmHg)	BMI (kg/m^2^)	HDL chol (mmol/l)	LDL chol (mmol/l)	Triglycerides (mmol/l)	hsCRP (mg/l)
**Group 1: aspirin 300** **n =9**	3 male 6 female	32.00 (30.00 - 37.00)	119 ± 6	82 ± 3	23.114 (20.91 - 26.50)	1.83 (1.32 – 2.34)	2.74 (2.39 - 3.50)	1.09 (0.81 - 1.59)	0.45 (0.1 – 0.90)
**Group 2: aspirin 75** **n = 9**	4 male 5 female	33.00 (29.99 - 41.00)	123 ± 6	82 ± 4	25.26 (22.18 - 28.13)	1.77 (1.30 – 1.99)	2.73 (2.22 - 3.38)	0.99 (0.69 - 1.57)	0.50 (0.40 – 1.00)
**Group 3: clopidogrel** **n = 9**	3 male 6 female	33.50 (32.00 - 43.75)	125 ± 4	84 ± 3	25.00 (20.43 - 28.67)	1.71 (1.45 – 2.12)	2.49 (2.26 - 3.14)	0.93 (0.77 - 1.53)	0.60 (0.20 – 0.93)
**Group 4: untreated** **n = 9**	4 male 5 female	37.00 (32.00 - 42.00)	124 ± 4	83 ± 2	24.11 (21.73 - 28.69)	1.66 (1.28 – 2.08)	2.52 (2.06- 3.32)	1.06 (0.73 – 1.64)	0.45 (0.28 – 0.65)
**Unvaccinated group** **n = 40**	16 male 24 female	32.75 (30.25 - 39.75)	122 ± 5	82 ± 4	22.80 (20.78 – 26.14)	1.88 (1.52 – 2.01)	2.41 (2.17 – 3.01)	1.01 (0.78 – 1.33)	0.77 (0.34 – 1.60)

Biometric data and cardiovascular risk profiles for the subjects in all groups are shown. Values are expressed as the mean ± SEM or median (IQR). SBP: systolic blood pressure; DBP: diastolic blood pressure; BMI: body mass index; HDL: high density lipoprotein; LDL: low density lipoprotein; hsCRP: high-sensitivity C-reactive protein.

The other 40 subjects were previously recruited into a clinical study designed to identify novel biomarkers of aspirin resistance in response to 28 days of treatment with aspirin 300 mg once daily [31]. The same markers as above were measured in stored serum samples, with the exception of TXB_2_, which had been previously assessed in urine samples. In addition to VCAM-1, further endothelial markers, including E-selectin and intercellular adhesion molecule-1 (ICAM-1) were also assessed in this population. hs-CRP was also measured in stored samples (Quintiles Drug Research Unit, London). Among previously measured parameters for this population, salicylate levels were available in a subpopulation of 22 individuals.

The clinical studies were reviewed and given favourable opinion by the NRES London—Dulwich Research Ethics Committee (ref. number 13/LO/1664; South London network study identification number 16644) and the Riverside Research Ethics Committee, London, UK (ref. number 07/Q0401/1) and registered on the UK Clinical Research Network Portfolio. All participants gave informed consent. The studies were performed conforming to the Declaration of Helsinki.

### Enzyme-linked immunosorbent assays

Enzyme-linked immunosorbent assays (ELISAs) were carried out to measure serum levels of netrin-1 (SEB827Hu, Cloud-Clone Corp., China), PGE_2_ (MBS007171, MyBioSource, Inc., USA), TXB_2_ (CSB-E08046h, Cusabio, China), VCAM-1 (DVC00, R&D Systems, UK), ICAM-1 (850.540.096, Diaclone, France) and E-selectin (CSB-E04540h, Cusabio, China). All kits were commercially available and used as per the manufacturer instructions. Inter-assay variability of all kits was found to be <5%.

### Western blot

Western blotting of serum samples was performed with both rat anti-netrin-1 and goat anti-netrin-1 primary antibodies as previously described by our group. Prior to use, serum was treated, as per previously reported methodology [32], for lipid, IgG and albumin depletion. Briefly, samples were centrifuged for 15 minutes at 15000 x *g* at room temperature to remove the lipid component; IgG depletion was subsequently performed on the delipidated serum with a Protein G Sepharose bead suspension (10278424, GE Healthcare Ltd, UK), and was followed by centrifugation in cold ethanol to remove the albumin-rich serum fraction [32].

Proteins were subsequently re-suspended in radioimmunoprecipitation assay (RIPA) buffer and measured by BCA assay. 10 μg of each protein sample was separated on a SDS-PAGE gel (10% acrylamide), and transferred to a polyvinylidene difluoride membrane. After 1 hour blocking in phosphate buffer saline containing 5% milk / 0.1% Tween-20, the membranes were probed with either rat anti-netrin-1 or goat anti-netrin-1 antibodies (both 1:100 in blocking solution; R&D System), for 2 hours at room temperature. After washing, membranes were incubated with goat anti-rat or donkey anti-goat secondary antibodies as appropriate (1:2000; Cell Signalling, UK). Bands were detected with enhanced chemiluminescence reagent on Hyperfilm (Amersham Biosciences, UK).

### Statistics

Statistical analyses were performed using GraphPad Prism (version 6.0) software. Parametric data are expressed as the mean ± standard error of mean (SEM), whilst non-parametric data are expressed as the median with interquartile ranges (IQR). ANOVA was used to compare percentage variation of each study variable between the 4 immunisation groups in the vaccine study. Baseline and post-treatment values were compared within each group using a paired parametric or non-parametric test as appropriate. Netrin-1 correlation with the other study variables was analysed by Spearman correlation test (since netrin-1 was not normally distributed). A p value of <0.05 was taken as statistically significant.

## Abbreviations

COX: cyclo-oxygenase
CRP: C-reactive protein
hsCRP: high sensitivity C-reactive protein
ICAM-1: intracellular adhesion molecule
PGE2: prostaglandin E2
TXB2: thromboxane B2
VCAM-1: vascular cell adhesion molecule-1

## References

[1] Ramesh G, Berg A, Jayakumar C (2011). Plasma netrin-1 is a diagnostic biomarker of human cancers. Biomarkers.

[2] Yildirim ME, Kefeli U, Aydin D, Sener N, Gumus M (2016). The value of plasma netrin-1 in non-small cell lung cancer patients as diagnostic and prognostic biomarker. Tumour Biol.

[3] Grandin M, Meier M, Delcros JG, Nikodemus D, Reuten R, Patel TR, Goldschneider D, Orriss G, Krahn N, Boussouar A, Abes R, Dean Y, Neves D (2016). Structural decoding of the netrin-1/UNC5 interaction and its therapeutical implications in cancers. Cancer Cell.

[4] Layne K, Ferro A, Passacquale G (2015). Netrin-1 as a novel therapeutic target in cardiovascular disease: to activate or inhibit?. Cardiovasc Res.

[5] van Gils JM, Derby MC, Fernandes LR, Ramkhelawon B, Ray TD, Rayner KJ, Parathath S, Distel E, Feig JL, Alvarez-Leite JI, Rayner AJ, McDonald TO, O'Brien KD (2012). The neuroimmune guidance cue netrin-1 promotes atherosclerosis by inhibiting the emigration of macrophages from plaques. Nat Immunol.

[6] Bouhidel JO, Wang P, Li Q, Cai H (2014). Pharmacological postconditioning treatment of myocardial infarction with netrin-1. Front Biosci (Landmark Ed).

[7] Zhang J, Cai H (2010). Netrin-1 prevents ischemia/reperfusion-induced myocardial infarction via a DCC/ERK1/2/eNOS s1177/NO/DCC feed-forward mechanism. J Mol Cell Cardiol.

[8] Passacquale G, Phinikaridou A, Warboys C, Cooper M, Lavin B, Alfieri A, Andia ME, Botnar RM, Ferro A (2015). Aspirin-induced histone acetylation in endothelial cells enhances synthesis of the secreted isoform of netrin-1 thus inhibiting monocyte vascular infiltration. Br J Pharmacol.

[9] Delloye-Bourgeois C, Goldschneider D, Paradisi A, Therizols G, Belin S, Hacot S, Rosa-Calatrava M, Scoazec JY, Diaz JJ, Bernet A, Mehlen P (2012). Nucleolar localization of a netrin-1 isoform enhances tumor cell proliferation. Sci Signal.

[10] Paradisi A, Maisse C, Bernet A, Coissieux MM, Maccarrone M, Scoazec JY, Mehlen P (2008). NF-kappaB regulates netrin-1 expression and affects the conditional tumor suppressive activity of the netrin-1 receptors. Gastroenterology.

[11] Hingorani AD, Cross J, Kharbanda RK, Mullen MJ, Bhagat K, Taylor M, Donald AE, Palacios M, Griffin GE, Deanfield JE, MacAllister RJ, Vallance P (2000). Acute systemic inflammation impairs endothelium-dependent dilatation in humans. Circulation.

[12] Donald AE, Charakida M, Cole TJ, Friberg P, Chowienczyk PJ, Millasseau SC, Deanfield JE, Halcox JP (2006). Non-invasive assessment of endothelial function: which technique?. J Am Coll Cardiol.

[13] Deanfield JE, Halcox JP, Rabelink TJ (2007). Endothelial function and dysfunction: testing and clinical relevance. Circulation.

[14] Hadi HA, Carr CS, Al Suwaidi J (2005). Endothelial dysfunction: cardiovascular risk factors, therapy, and outcome. Vasc Health Risk Manag.

[15] Vlachopoulos C, Xaplanteris P, Sambatakou H, Mariolis E, Bratsas A, Christoforatou E, Miliou A, Aznaouridis K, Stefanadis C (2011). Acute systemic inflammation induced by influenza A (H1N1) vaccination causes a deterioration in endothelial function in HIV-infected patients. HIV Med.

[16] Fontana P, Nolli S, Reber G, de Moerloose P (2006). Biological effects of aspirin and clopidogrel in a randomized cross-over study in 96 healthy volunteers. J Thromb Haemost.

[17] Di Francesco L, Totani L, Dovizio M, Piccoli A, Di Francesco A, Salvatore T, Pandolfi A, Evangelista V, Dercho RA, Seta F, Patrignani P (2009). Induction of prostacyclin by steady laminar shear stress suppresses tumor necrosis factor-alpha biosynthesis via heme oxygenase-1 in human endothelial cells. Circ Res.

[18] Birukova AA, Zagranichnaya T, Fu P, Alekseeva E, Chen W, Jacobson JR, Birukov KG (2007). Prostaglandins PGE(2) and PGI(2) promote endothelial barrier enhancement via PKA- and Epac1/Rap1-dependent Rac activation. Exp Cell Res.

[19] Razandi M, Pedram A, Rubin T, Levin ER (1996). PGE2 and PGI2 inhibit ET-1 secretion from endothelial cells by stimulating particulate guanylate cyclase. Am J Physiol.

[20] Eskildsen MP, Hansen PB, Stubbe J, Toft A, Walter S, Marcussen N, Rasmussen LM, Vanhoutte PM, Jensen BL (2014). Prostaglandin I2 and prostaglandin E2 modulate human intrarenal artery contractility through prostaglandin E2-EP4, prostacyclin-IP, and thromboxane A2-TP receptors. Hypertension.

[21] Kalinski P (2012). Regulation of immune responses by prostaglandin E2. J Immunol.

[22] van Gils JM, Ramkhelawon B, Fernandes L, Stewart MC, Guo L, Seibert T, Menezes GB, Cara DC, Chow C, Kinane TB, Fisher EA, Balcells M, Alvarez-Leite J (2013). Endothelial expression of guidance cues in vessel wall homeostasis dysregulation under proatherosclerotic conditions. Arterioscler Thromb Vasc Biol.

[23] Ly NP, Komatsuzaki K, Fraser IP, Tseng AA, Prodhan P, Moore KJ, Kinane TB (2005). Netrin-1 inhibits leukocyte migration *in vitro* and *in vivo*. Proc Natl Acad Sci U S A.

[24] Layne K, Di Giosia P, Ferro A, Passacquale G (2016). Anti-platelet drugs attenuate the expansion of circulating CD14highCD16+ monocytes under pro-inflammatory conditions. Cardiovasc Res.

[25] Smyth EM, Grosser T, Wang M, Yu Y, FitzGerald GA (2009). Prostanoids in health and disease. J Lipid Res.

[26] Conary JT, Parker RE, Christman BW, Faulks RD, King GA, Meyrick BO, Brigham KL (1994). Protection of rabbit lungs from endotoxin injury by *in vivo* hyperexpression of the prostaglandin G/H synthase gene. J Clin Invest.

[27] Bouchard JF, Chouinard J, Lamontagne D (2000). Participation of prostaglandin E2 in the endothelial protective effect of ischaemic preconditioning in isolated rat heart. Cardiovasc Res.

[28] Chen M, Divangahi M, Gan H, Shin DS, Hong S, Lee DM, Serhan CN, Behar SM, Remold HG (2008). Lipid mediators in innate immunity against tuberculosis: opposing roles of PGE2 and LXA4 in the induction of macrophage death. J Exp Med.

[29] Lima J, Siqueira M, Pedro T, Ponte C, Peres L, Marinho S, Castello-Branco LR, Antas PR (2015). The role of host soluble inflammatory mediators induced by the BCG vaccine for the initiation of *in vitro* monocyte apoptosis in healthy Brazilian volunteers. J Inflamm (Lond).

[30] Ranganathan PV, Jayakumar C, Mohamed R, Dong Z, Ramesh G (2013). Netrin-1 regulates the inflammatory response of neutrophils and macrophages, and suppresses ischemic acute kidney injury by inhibiting COX-2-mediated PGE2 production. Kidney Int.

[31] Floyd CN, Goodman T, Becker S, Chen N, Mustafa A, Schofield E, Campbell J, Ward M, Sharma P, Ferro A (2014). Increased platelet expression of glycoprotein IIIa following aspirin treatment in aspirin-resistant but not aspirin-sensitive subjects. Br J Clin Pharmacol.

[32] Fu Q, Bovenkamp DE, Van Eyk JE (2007). A rapid, economical, and reproducible method for human serum delipidation and albumin and IgG removal for proteomic analysis. Methods Mol Biol.

